# Beard Alopecia: An Updated and Comprehensive Review of Etiologies, Presentation and Treatment

**DOI:** 10.3390/jcm12144793

**Published:** 2023-07-20

**Authors:** Michael Kaiser, Rama Abdin, Marita Yaghi, Simonetta I. Gaumond, Joaquin J. Jimenez, Naiem T. Issa

**Affiliations:** 1 Dr. Phillip Frost Department of Dermatology and Cutaneous Surgery, University of Miami Miller School of Medicine, Miami, FL 33136, USA; mxy537@med.miami.edu (M.Y.); sxg1204@miami.edu (S.I.G.);; 2 Charles E. Schmidt College of Medicine, Florida Atlantic University, Boca Raton, FL 33431, USA; 3 Forefront Dermatology, Vienna, VA 22182, USA; 4 Issa Research and Consulting, LLC, Springfield, VA 22152, USA

**Keywords:** beard, alopecia, alopecia areata

## Abstract

Facial hair is an important social and psychologic aspect of clinical appearance for men. The purpose of this review is to provide a comprehensive overview of the causes of alopecia of the beard including the prevalence, pathophysiology, clinical presentation, and treatment. In this review, we highlight more common causes of beard alopecia including alopecia areata and pseudofolliculitis barbae, infectious causes such as tinea barbae and herpes simplex folliculitis, and rare causes including dermatopathia pigmentosa reticularis and frontal fibrosing alopecia. This review serves as an important resource for clinicians when faced with patients suffering from beard alopecia.

## 1. Introduction

Male facial hair patterning is varied amongst ethnicities and plays an important role in male individualism and masculinity. The androgenetic effects of facial hair are predominantly controlled by genetics [1,2]. Male facial hair has been shown to play a significant role in perceived attractiveness and was associated with increased long-term attractiveness [1]. The important social and individual importance of the male beard may be compromised by beard alopecia. This includes trauma, endocrinopathies, autoimmune or systemic processes, burns, and infectious etiologies [3,4]. Early recognition and management of beard alopecia can alleviate potentially psychologically damaging facial hair loss. However, comprehensive literature on beard alopecia and related clinical approaches remains sparse. Herein, we focus on reviewing common and rare etiologies of beard loss, with a focus on presentation, diagnosis, and treatment modalities.

## 2. Inflammatory Causes of Alopecia

### 2.1. Alopecia Areata

Alopecia areata (AA) is an autoimmune disorder caused by T-cell-mediated destruction of hair follicles resulting in well-circumscribed patches of nonscarring alopecia involving any part of the body [5]. The two most common sites are the scalp and the beard, with the latter accounting for ~28% of cases and commonly referred to as alopecia areata barbae (AAB) [5,6]. Trichoscopic findings of AAB include small black dots, yellow dots, exclamation mark hairs, short vellus hairs, tapering hairs, and nonfollicular white dots [7,8]. Biopsy specimens in the acute phase typically show peribulbar inflammatory infiltrate, mostly CD4+ and CD8+ T cells, within the subcutaneous hair follicles. There is also a significant decrease in the anagen-to-telogen hair ratio, with an overall decrease in the number of terminal hairs. Histologic findings of chronic AA include variable lymphocytic infiltrate surrounding miniaturized hairs of the papillary dermis, with or without fibrosis of the follicle [9]. Two studies assessing AAB epidemiology found the mean age of onset to be between 30–40 years old [6,7]. The recurrence rate has been reported between 15 and 25.5% [6,7]. AAB may also have associations with disease states such as diabetes mellitus, vitiligo, hypothyroidism, and vitamin D deficiency and may be secondary to inflammatory stress from infections such as COVID-19 [6,10,11,12,13].

No established treatment algorithm currently exists as no randomized controlled trials have been conducted with therapeutics for AAB. Nonetheless, numerous treatments are promising. Topical and intralesional steroids are the most common primary treatment modalities for AAB [6]. Intralesional triamcinolone (2.5 mg/mL) is commonly used for AAB [14]. Systemic corticosteroids have shown favorable results in treating AA with one study reporting nearly 70% hair regrowth [15]. Topical minoxidil may have a role in hair regrowth in AAB [5,16]. *Pototschnig* et al. reported a case of successful treatment of AAB with three injections of platelet-rich plasma every six weeks [17]. Topical immunotherapy with 2,3 -diphenylcyclopropenone (DPCP) has been used anecdotally with the authors noting positive results in beard regrowth, although no published literature is available [18,19]. Furthermore, targeted treatments for autoimmune conditions such as alopecia areata represents a potentially exciting new therapy [20]. Janus kinase (JAK) inhibitors such as oral tofacitinib and ruxolitinib, which have been effective in severe AA forms such as alopecia universalis and in patients with treatment-resistant AA, have been shown to nearly completely restore facial hair in men with AAB [21,22,23]. Topical JAK inhibitors are also currently undergoing phase II and phase III clinical trials with favorable results [24,25]. Specifically, the combination of 5–10 mg BID of tofacitinib and 3.5 mg BID oral minoxidil has been shown to be efficacious in treating alopecia areata [26,27]. In cases of vitamin D deficiency-induced alopecia areata, topical calcipotriol, a vitamin D analog, has shown promising results, but further investigation is needed to fully characterize the role and mode of vitamin D supplementation in the treatment of AA [28,29]. Given these findings, a treatment approach to alopecia areata of the beard can be extrapolated from the treatment algorithm of scalp alopecia areata which includes topical or intralesional corticosteroids in mild to moderate cases and JAK inhibitors in severe or treatment-resistant cases. Patients may also benefit from combination therapy with oral or topical minoxidil. Topical immunotherapy or platelet-rich plasma injections may also be considered if the first-line options do not produce favorable results. As always, a thorough history must be obtained to create a patient-centered treatment approach that addresses any underlying disorders that may complicate or worsen alopecia areata, including vitamin D deficiency.

### 2.2. Pseudofolliculitis Barbae

Pseudofolliculitis barbae (PFB) is a chronic inflammatory process affecting the perifollicular and follicular skin of the face. Asian and African men are most commonly affected due to the morphology of the hair within the follicle [30]. It is estimated that nearly 5 million Black patients in the United States suffer from PFB [30]. In PFB patients, the hair shaft is tightly coiled and has an elliptical shape, thus having a propensity to penetrate into the epidermis as the hair follicle has a curved shape [31]. This penetration stimulates a foreign body reaction causing pruritis and inflammation with papule/pustule formation [31]. Shaving or plucking the hair is known to potentiate this inflammatory process. The shaved hair is sharp and situated close to or into the epidermis, leading to downward growth and subsequent epidermal penetration. PFB often affects areas of the body that are routinely shaved, including the face and neck area in men, but can also affect women, especially those with hirsutism [31]. Inflammation can lead to pustules, micro-abscesses, secondary bacterial infection, post-inflammatory hyperpigmentation, hypertrophic scarring, keloid formation, and cicatricial hair loss of the beard area [32,33]. Trichoscopic findings include perifollicular and follicular papules and pustules as well as perifollicular hyperkeratosis. Trichoscopy may also show either sharp or blunt hairs depending on the method of shaving [30]. Histology demonstrates polymorphonuclear cell infiltration surrounding the infiltrating shaft leading to epidermal micro-abscesses and, in more severe cases, foreign body granulomatous inflammation [30].

Treatment of PFB is multifaceted and should begin immediately to prevent potentially permanent scarring. One preventative measure is to cease shaving in the affected area to allow hair to grow. However, it is important to note that in populations such as military recruits or females, this may not be a viable option. Altering shaving practices is essential to improving PFB in patients. Hair clippers are preferred over razor blades to keep hair length greater than 0.5 mm [34]. This prevents both extrafollicular and transfollicular penetration of the hair which occurs in a close shave from razor blades. Foil guards or metal protectors can be used on razors to achieve a closer shave while maintaining a longer hair shaft length [35]. Patients with PFB are advised to shave in a superior-to-inferior direction without applying additional tension to the skin [36]. Prior to shaving, washing the area with warm water and a mild cleanser can soften and even release embedded hairs. Maintaining beard softness can be achieved through the application of warm compresses daily and brushing the beard [37]. Treatment of PFB consists of avoiding shaving in areas with active pustules, topical clindamycin, benzoyl peroxide, oral antibiotics, and keratolytics such as retinoids and salicylic acid [30]. Topical therapies such as corticosteroids and retinoids are first-line treatments for PFB, acting as keratinolytics and further reducing irritation [38,39]. These therapies are advantageous in their ease of use and relative cost-effectiveness. Definitive treatment can be achieved with depilation through electrolysis or laser hair removal, but these options may pose a financial burden [34].

### 2.3. Folliculitis Decalvans

Folliculitis decalvans (FD) is an inflammatory condition leading to cicatricial alopecia. The underlying cause of FD is not fully understood, but *Staphylococcus aureus* bacteria may play a role in its pathogenesis given that it has been isolated in most cases of untreated FD [40]. Clinically, FD usually affects the vertex and occipital regions of the scalp, with the initial lesion presenting as an erythematous follicular papule [40]. The characteristic findings are areas with scarring and follicular pustules [40]. FD can present with pruritis, trichodynia, yellow crusting, scale, and pustules [41,42]. Trichoscopic findings include perifollicular erythema and scale, follicular hyperkeratosis, pustules, tufted hairs, and white dots [41,43]. Histologically, FD is characterized as having a prominent neutrophilic infiltrate contained in the intrafollicular and perifollicular space. As the disease progresses, the inflammatory process extends into the dermis and ultimately leads to periadnexal dermal fibrosis [42]. Advanced lesions have been shown to also contain hair shaft granulomas and fibrous tracts replacing hair follicles [41]. Treatment consists of oral antibiotics, with extended courses of clindamycin with rifampicin showing the lowest rates of relapse. Doxycycline and azithromycin have also been shown to be good alternative treatments, albeit with shorter relapse rates ranging from 3 to 6 months [44]. Oral isotretinoin has also been shown to treat FD and prevent disease recurrence for the longest period [45]. The treatment protocol suggested by *Miguel-Gómez* et al. is an appropriate initial approach in the management of FD. For mild–moderate cases, a combination of topical corticosteroids and topical antibiotics is the basis of treatment. Topical tacrolimus and intralesional corticosteroids may be added in certain cases or cases associated with inflammation. Oral antibiotics such as tetracyclines for 8–12 weeks, or azithromycin in cases of resistance, are useful for inflammatory flare-ups. Fusidic acid and trimethoprim–sulfamethoxazole may also be used. For severe cases, local treatment options are the same as in mild–moderate cases; however, oral therapy involves a combination of antibiotics such as rifampicin and clindamycin at higher and more frequent dosing for 10 weeks as well as oral steroids, isotretinoin, and dapsone. Although antibiotics are the basis of the initial treatment approach, it is important to consider the potential for antibiotic resistance in relapsing cases. Photodynamic therapy can also be used as complementary treatment in some cases, but it has been associated with pain in some cases [42]. Recently, subcutaneous adalimumab at a starting dose of 160 mg, with eventual step-down in strength, has been used successfully in treatment-resistant cases with no major side effects, which presents a potential future treatment option [46].

Cases of FD affecting the beard have been documented, albeit rarely [43,47]. *Senatore* et al. published one of the first cases to highlight the exclusive involvement of the beard in a patient with FD. The patient was a 45-year-old man who presented with bilateral erythematous pustular lesions on the cheeks affecting the beard. There were prominent areas of scarring alopecia in the beard area, and treatment consisted of oral doxycycline 200 mg daily with topical antiseptic treatment resulting in resolution without progression at the four-month follow-up visit [43]. This appears to be in concordance with the above-proposed treatment protocol, suggesting that FD of the beard can likely be treated in the same manner as scalp FD.

### 2.4. Frontal Fibrosing Alopecia

Frontal fibrosing alopecia (FFA) is a scarring alopecia that primarily affects post-menopausal women, but men may also be affected [48]. Clinically, FFA presents as a progressive hairline recession in the frontotemporal and eyebrow regions [48]. On histology, FFA appears as a lichenoid perifollicular lymphocytic infiltrate progressing to a loss of hair follicles and the formation of fibrous tracts that replace hair follicles [49]. There is debate as to whether FFA is a variant of lichen planopilaris or a distinct pathologic process. While the incidence is unknown, cases of FFA have been increasing in frequency [49]. The underlying cause of FFA is not known, but it is thought that loss of immune privilege in the hair follicle due to IFN-γ release stimulates Th1-mediated destruction and fibrosis of the bulge region of the hair follicle [50]. While men are less commonly affected, FFA can present with loss of sideburns and the beard area [50,51]. In a recent multicenter study by *Pathoulas* et al., one in five patients reported a loss of beard hair at FFA onset [52]. Trichoscopic findings of FFA include perifollicular erythema, lonely hair sign, loss of vellus hairs, follicular hyperkeratosis, and loss of hair follicle openings [50,51]. Treatments of FFA include potent topical steroids and calcineurin inhibitors which can reduce inflammation but may not slow the progression of alopecia [50]. Systemic therapy is most effective, with oral prednisone stopping progressive hair loss, but relapse is common after stopping therapy. Hydroxychloroquine has also been shown to be effective in preventing progressive alopecia, but it may not be a suitable option in patients with underlying retinopathy or certain hematologic disorders [48,51,53]. Intralesional triamcinolone has been shown to stabilize and improve FFA [50]. It is important to watch for skin atrophy if high and/or multiple doses are used [53]. An observational study by Pindado-Ortega found that 5% topical minoxidil and 0.05% clobetasol solution therapy in combination with 1–7 capsules per week of 0.5 mg oral dutasteride is superior to combination with other systemic therapies such as 2.5–5 mg daily finasteride, 200–400 mg hydroxychloroquine daily, doxycycline 100 mg daily, and 5–20 mg isotretinoin daily. It is important to note the small number of patients prescribed the alternative systemic therapies in this study. Overall, combination treatment is the most prevalent treatment approach. In the beginning stages, or if symptoms include pruritis, topical steroids and non-steroidal alternatives (e.g., calcineurin inhibitors) are used. *Iorizzo* and *Tosti* note that topical minoxidil may be used to treat FFA due to its antifibrotic properties which may thwart scarring and/or address concurrent androgenetic alopecia [53]. Therefore, treatment of FFA involves the evaluation of possible concurrent disorders, commonly androgenetic alopecia, to devise a personalized treatment plan. Severe cases may benefit from systemic corticosteroids as well as other immunomodulators. Prognosis is variable, and it is possible that patients will experience continued hair recession even with treatment, albeit at a slower rate [49]. Future treatment options, for which clinical trials are underway, may include Janus kinase inhibitors, including ritlecitinib (NCT05549934) and platelet-rich plasma (NCT03335228).

### 2.5. Pseudopalade of Brocq

Pseudopalade of Brocq (PPB) is a slowly progressing cicatricial alopecia characterized by a lymphocytic infiltrate with an unknown pathogenesis [54]. The incidence of PPB is estimated to range from 3.0 to 7.3% in the general population [55,56]. PPB clinically appears as multiple patches of alopecia on the scalp that can enlarge to form plaques with irregular borders [57]. There is debate about whether PPB is a unique disease process (primary PPB) or if it is the final scarring stage of other autoimmune diseases including discoid lupus or lichen planopilaris (secondary PPB) [57]. Early PPB presents with localized erythema but is often seen as late-stage atrophy clinically [57]. Trichoscopic features are nonspecific but include a lack of follicular openings, white areas, and dystrophic hairs at lesion edges [58]. Histopathology of PPB is often nondiagnostic but includes evidence of scarring, an absence of inflammation, minimal follicular plugging, and columns of fibrosis [59,60]. There is often a perifollicular lymphocytic infiltrate confined to the upper two-thirds of the hair follicle [61]. Management of PPB is difficult, with a poor response to intralesional and systemic steroids. Treatment with oral hydroxychloroquine, isotretinoin, or mycophenolate mofetil can improve the condition with varying results [61]. There is no consensus on treatment, and clinicians should trial the above-mentioned medications in an attempt to achieve remission in this disease with often unpredictable prognosis.

PPB usually affects the scalp area but can rarely affect the beard. *Madani* et al. outlined a case of PPB in a 34-year-old male involving the scalp and beard [60]. The patient presented with a two-month history of hair loss in the scalp and beard where oval patches of scarring alopecia were identified. Biopsies of both areas showed late-stage scarring alopecia with a negative direct immunofluorescence. The patient had no other skin lesions and no features of another autoimmune disease. The patient was treated with a combination of 200 mg of hydroxychloroquine twice daily, topical clobetasol ointment daily, and monthly intralesional triamcinolone acetonide (10 mg/mL). Although the patient developed two more areas of scalp alopecia during the 6 documented months of therapy, it is possible that a longer treatment regimen is needed for this condition, or that the addition of other immunomodulators is required for adequate resolution.

### 2.6. Seborrheic Dermatitis

Seborrheic dermatitis (SD) is a disease caused by environmental and intrinsic factors such as colonization with *Malassezia* species, alterations in sebaceous secretions, and individual susceptibility based on genetic factors [62]. SD commonly affects the scalp, face, and upper chest [62]. SD often involves areas of the skin with increased sebaceous gland production of sebum such as the face, upper chest, and scalp. An estimated 3–5% of young adults are affected by SD each year, with men usually more commonly affected than women [63]. Additionally, there have been correlations with seborrheic dermatitis affecting the areas of the face with increased basal temperatures such as the nasolabial folds, beard, and mustache [64]. SD that affects the face can lead to hair shedding and hair thinning. Clinical signs of SD include erythema, scaling, and itching of the affected area. Progression of the disease can lead to telogen effluvium, thinning of the hair shaft, and reduced anagen hairs [65]. Trichoscopic findings of SD include thin arborizing vessels, atypical red vessels, and a yellow scale [66].

Topical antifungal agents, including ketoconazole and bifonazole, are some of the most common agents used to treat SD. Additionally, some studies have demonstrated that topical steroids including hydrocortisone and betamethasone used alone or in conjunction with topical antifungals can also treat SD [67]. Short-term use of topical corticosteroids is preferred as long-term use can lead to atrophy of the skin and hypertrichosis [67]. Other therapeutic options for SD include oral antifungals such as ketoconazole and itraconazole, and topical calcineurin inhibitors such as pimecrolimus and tacrolimus as well as systemic therapy with oral isotretinoin [68,69]. Recently, the use of tapinarof cream 1% daily has been successful as well [70].

## 3. Mechanical Causes of Alopecia

### 3.1. Trichotillomania

Trichotillomania is classified as an obsessive–compulsive and related disorder in the latest *Diagnostic and Statistical Manual of Mental Disorders V* (DSM-V). Prevalence in the United States is estimated to be 0.6% [71,72]. The average age of onset is usually between 10 and 13 years old, with the scalp and eyebrows being the most common areas affected [72]. Trichotillomania has two different associated methods of hair removal named automatic pulling and focused pulling. Automatic pulling occurs when patients pluck hairs without recognizing their actions. Focused pulling is performed by an individual who is aware of their actions and is often induced by stress or anxiety [73]. Dermoscopy remains one of the best ways to diagnose true trichotillomania. Characteristic dermoscopic findings include short vellus hairs, reduced hair density, hair powder, flame hairs, hair splitting, upright regrowing hairs, V-sign, and the hallmark finding of broken hairs with different shaft lengths [74]. Biopsy specimens show evidence of follicular destruction secondary to external injury including hair follicle anatomy deformities, perifollicular and intrafollicular hemorrhage, hair shaft loss, trichomalacia, and melanin pigment casts [75]. Trichotillomania affecting the beard is a rare finding but has been reported. One case reports of a 14-year-old boy presenting with trichotillomania affecting the beard with dermoscopic findings of perifollicular hemorrhage with a “pluck out sign” [73]. Management of trichotillomania includes habit reversal therapy, selective serotonin reuptake inhibitors (SSRIs), and clomipramine [76].

### 3.2. Traction Alopecia of the Beard

Traction alopecia (TA) is due to traumatic styling or grooming with excessive pulling leading to both reversible alopecia as well as irreversible cicatricial alopecia [77]. It is difficult to estimate the exact prevalence of traction alopecia as it varies greatly within and between populations due to variations in hair styling practices [77]. A history of excessive tension is often elucidated when questioning the patient about grooming habits. Hairstyles such as tight buns, weaves, and braids, as well as grooming for religious reasons in Sikh and Jewish males, can lead to TA. TA often presents as sudden onset of patchy bilateral, symmetric hair loss with associated perifollicular erythema or papules in affected areas of the face and scalp [77]. Early TA is characterized by localized folliculitis in scalp areas under increased tension and can progress to scarring alopecia with decreased follicular markings over time [77]. Additionally, the “fringe sign”, or retained hairs along the frontal rim ahead of the point of tension, is a finding seen in both early and late TA [78]. Excessive tension causes tenting of the hair follicle and is an indicator of the disease process [79]. Trichoscopic findings include decreased hair density with a lack of follicular openings, lack of hairs with pigmented follicular openings, black dots, and broken hairs at various lengths [77]. On histology, there is an increased number of telogen and catagen hairs, preserved sebaceous glands, and decreased terminal hairs [78]. Longstanding TA can show decreased terminal follicles that have been replaced with fibrotic tracts [78].

It is important to inquire about cultural and religious grooming procedures as some may contribute to TA of the beard. For example, Sikh males are not to cut their hair given their religious beliefs. Thus, the grooming practice amongst this community is to tie the hair in a tight knot. As a result, TA affecting the submandibular area is sometimes seen in Sikh men [80]. Management of TA consists of stopping the offending hair practice when possible to reduce follicular tension and recommending styling practices that create less tension. In more severe cases, intralesional corticosteroids combined with oral or topical antibiotics are beneficial [79]. Hair follicle transplantation can also provide cosmetic benefit but may not be the most cost-effective approach [81].

## 4. Infectious Causes of Alopecia

### 4.1. Tinea Barbae

Tinea barbae is a rare complication caused by dermatophytes such as *Trichophyton rubrum*, *Trichophyton violaceum, Trichophyton tonsurans*, *and Microsporum canis* [82]. Dermatophytes invade structures containing keratin such as skin, nails, and hair [83]. Tinea barbae often presents with an inflammatory form consisting of a classic presentation of a kerion, which is a tender, oozing plaque with associated draining sinuses [83]. Deep folliculitis due to infectious invasion is termed sycosis barbae and can be caused by *S. aureus* bacteria or tinea fungal infections [84]. Affected beard hair becomes brittle and easily removed. Electron microscopy also reveals dermatophyte invasion of hair cortex cells and disruption of the anagen growth apparatus [82]. A noninflammatory form of tinea barbae also exists and is a more superficial infection typically characterized by erythematous plaques and associated papules/pustules [83]. Trichoscopic findings of tinea capitis, which can likely be extrapolated to tinea barbae, include corkscrew hairs, comma hairs, zigzag hairs, bent hairs, broken hairs, black dots, and morse code-like hairs in addition to perifollicular scaling [85]. Biopsy specimens should be prepared with periodic acid–Schiff staining as fungal features may not be evident with hematoxylin and eosin staining. Histology of general tinea infection will show fungal hyphae, arthroconidia, and inflammation within the dermis and epidermis [83]. Treatment of tinea barbae is accomplished with antifungal agents such as terbinafine or itraconazole [86]. *T. rubrum* can be treated with oral itraconazole 200 mg per day for up to 30 days if there is nail involvement [87]. Systemic treatment regimens for tinea barbae include griseofulvin 500 mg daily for 8 weeks, ketoconazole 200 mg daily for 8 weeks, itraconazole 200 mg daily for 8 weeks, or terbinafine 250 mg daily for 6 weeks [88]. *M. canis* and *T. tonsurans* are commonly treated with topical terbinafine cream 1%, oral terbinafine, or oral itraconazole [89]. Usually, after sustained treatment, there is complete resolution; however, hypertrophic scarring can lead to subsequent long-term hair loss [86].

### 4.2. Syphilitic Alopecia

Syphilis is a sexually transmitted infection caused by the spirochete *Treponema pallidum* [90]. Syphilis is separated into three stages, with primary syphilis presenting clinically as a painless chancre appearing at or near the site of infection within three weeks from first exposure [91]. Progression to secondary syphilis occurs when primary syphilis goes untreated and the spirochete disseminates through the blood to other parts of the body. Signs of secondary syphilis often include fever; lymphadenopathy; diffuse red-brown symmetric, non-pruritic rash affecting the palms and soles; and condyloma lata affecting the genitals or perineum [91,92]. The diagnosis of secondary syphilis can be made through the presence of the typical rash and a positive serologic Venereal Disease Research Laboratory (VDRL) test [92]. Rarely, syphilitic alopecia (SA) can appear as a manifestation of secondary syphilis occurring in 3–7% of patients [93].

There are two main patterns of alopecia associated with syphilis, namely symptomatic alopecia and essential alopecia. Symptomatic alopecia presents with the skin lesions of secondary syphilis in conjunction with diffuse or patchy alopecia of the scalp [94]. The “moth eaten” form is the most common presentation of SA and is considered pathognomonic of secondary syphilis and often affects the parietal and occipital regions of the scalp [94,95]. Trichoscopy reveals a reduced number of terminal hairs and the ostia lacking hair or even containing very small terminal vellus hairs with a diameter of less than 20 microns [93]. The cause of essential alopecia is local loss of capillaries in the area of alopecia and is devoid of any other clinical signs of syphilis infection [96]. Essential alopecia can cause nonscarring hair loss of the scalp but can also affect other regions of the body [94]. Additional findings in alopecic areas include abnormal red-brown pigmentation, follicular hyperkeratosis, dilated vessels, and colonization of the hair follicle with treponema pallidum [96]. Histopathologic evaluation of areas of SA often reveals a normal epidermis with regions of follicular hyperkeratosis, decreased anagen hair follicles, and increased catagen and telogen follicles [97].

SA affecting the beard area has been reported. *Piraccini* et al. presented one patient with a patch of SA affecting the mustache and another patient presenting with SA of the beard [93]. An area of “moth eaten” alopecia measured 2 mm in diameter affecting the mustache area. The other patient presented with a 3 mm area affecting the beard. In both patients, there were no changes in pigmentation in the affected areas. Trichoscopy further demonstrated follicles devoid of terminal hairs. Diagnosis of SA includes comprehensive patient history, pertinent skin findings of secondary syphilis, trichoscopic evaluation, and confirmatory VDRL blood test [93]. Diagnostic clues can be obtained through a full body skin exam for other cutaneous manifestations of secondary syphilis such as maculopapular eruption, papulosquamous rash affecting palms and soles, and condyloma lata [98]. A positive anti-treponemal antibody test and positive rapid plasma reagen tests are also important diagnostic clues [98]. Biopsy for syphilitic alopecia can also be diagnostic, showing spirochetes in the hair follicle [99]. All areas of alopecia resolved with the standard syphilis treatment of intramuscular injection of 2.4 million units of benzathine benzylpenicillin once a week for two consecutive weeks. Trichoscopy of the alopecic areas after antibiotic treatment demonstrated regrowing terminal hairs.

### 4.3. Herpetic Folliculitis/Sycosis

Herpes simplex virus (HSV) is a common cause of skin infection, and an estimated 40–63% of adults are seropositive for HSV-1 [100]. HSV-1 causes a variety of mucocutaneous infections including eczema herpeticum, keratoconjunctivitis, herpes labialis, and gingivostomatitis [101]. Herpetic folliculitis is an uncommon manifestation. It initially presents with localized burning, paresthesia, and pruritis antecedent to a rapidly progressive erythematous and papular patch [101]. However, an antecedent prodrome may not necessarily be present as in the case reported by *Al-Dhafiri* et al. where a 24-year-old male experienced herpetic folliculitis of the beard area that spread to the jaw, chin, and upper neck [102]. He further developed umbilicated vesicles and pustules on an erythematous base, canonical of cutaneous HSV infection, as the eruption progressed. Tzanck prep of a pustule demonstrated epithelial ballooning and multinucleated giant cells confirming the diagnosis of herpes virus infection. *Izumi* et al. also presented two cases of herpetic folliculitis of the beard with histopathology revealing HSV infection involving the hair follicle surface epithelium and root sheath with localized destruction of the follicle [101]. In addition to the typical herpes virus-associated cellular changes, histology revealed perifollicular and perivascular infiltrate extending through the dermis into the subcutaneous fat. The most accurate diagnostic technique is HSV viral polymerase chain reaction (PCR) [103]. A recent observational study found that there are no characteristic trichoscopic findings associated with herpetic folliculitis; however, the diagnostic value of dermoscopy could not be determined given the small sample size [104]. Treatment with antiviral medications such as valacyclovir and famciclovir leads to improvement [102].

## 5. Oncologic Causes of Alopecia

### Cutaneous T-Cell Lymphoma (CTCL)

Cutaneous T-cell lymphomas (CTCLs) are a group of disorders in which abnormal T lymphocytes infiltrate various levels of the skin, leading to flat patches, thin plaques, or nodules/tumors [105]. One of the most common forms of CTCL is mycosis fungoides (MF), which commonly presents clinically in older adults as multiple cutaneous patches, plaques, or tumors as well as generalized erythroderma [106]. Presentation is often confused with severe atopic dermatitis. The plaques of MF predominantly affect areas of skin that are usually shielded from sunlight such as the buttocks and medial thighs [107]. Other manifestations of MF include localized skin atrophy with telangiectasias and moderate hyperpigmentation as well as localized alopecia [106]. The cells of MF are atypical T cells expressing antigens such as CD2, CD4, and CD7 [106]. Sézary syndrome is a more aggressive form of MF that consists of diffuse epidermal involvement with atypical T cells circulating in peripheral blood [107]. The T cells circulating in Sézary syndrome are characterized by large cerebriform nuclei, and one of the diagnostic criteria for Sezary syndrome is ≥1000 of these abnormal T cells circulating in peripheral blood [108].

Alopecia has been characterized as a rare complication of MF. In a review of 1550 patients with MF/SS, 38 patients demonstrated some form of alopecia, with 34% of those patients having alopecia that appeared identical to alopecia areata, including 3 patients with beard alopecia [105]. Trichoscopic findings of folliculotropic mycosis fungoides are heterogeneous; these include decreased follicular openings, milky-white globules, yellow dots possibly with central black dots/broken hair, structureless patches, perifollicular white or hyperpigmented halos, short fine vessels, perifollicular scaling, and background discoloration [109,110,111,112]. Management of MF depends on the disease stage, but therapies include topical steroids, topical mechlorethamine, topical or oral bexarotene, phototherapy with UV A or B, total skin electron beam therapy, and local radiotherapy [113].

Alopecia mucinosa, commonly referred to as follicular mucinosis, is hypothesized to involve the stimulation of follicular keratinocytes by T-cell cytokines to deposit mucin in hair follicles and sebaceous glands [114]. Primary (idiopathic) follicular mucinosis is characterized by hypopigmented eczematous plaques and follicular papules, with or without alopecia and pruritis. It may present as a solitary lesion, typically on the head and neck, or multiple lesions may be present [114,115]. Histopathology of follicular mucinosis exhibits varying amounts of mucin deposit with lymphocytic infiltrate, which may be predominantly eosinophilic, in the sebaceous gland and outer root sheath. When associated with cutaneous T-cell lymphoma, findings include focal epidermotropism of lymphocytes between hair follicles and Pautrier micro-abscesses [114,115,116]. Trichoscopic findings include empty follicular openings with follicular plugs consisting of yellow-brown material [58]. *Bi* et al. describe alopecia areata-like hair loss of the beard with associated indurated plaques in a patient with biopsy-proven mycosis fungoides elsewhere on the body [105]. Although spontaneous resolution of alopecia is possible, there is no single effective treatment for primary follicular mucinosis. Reported treatment options include topical and intralesional corticosteroids, hydroxychloroquine, topical and oral bexarotene, topical antibiotics, dapsone, indomethacin, imiquimod, pentoxifylline, minocycline, ultraviolet light, and nitrogen mustard [114,117,118,119].

## 6. Rare/Genetic Causes of Alopecia

### Dermatopathia Pigmentosa Reticularis

Dermatopathia pigmentosa reticularis (DPR) is a rare pigmentary disorder that classically presents with a triad of widespread reticulate hyperpigmentation of the neck, trunk, and limbs with variable palmoplantar involvement, non-cicatricial alopecia, and onychodystrophy. It has been associated with adermatoglyphia, hypohidrosis or hyperhidrosis, palmoplantar hyperkeratosis, nonscarring blisters on the dorsa of the hands and feet, hyperpigmentation of the oral mucosa, bulbar conjunctiva and areolas, and hair sparsity affecting the eyebrows, pubis, and axillae [120,121].

With very few instances reported in the literature worldwide, knowledge of DPR pathophysiology remains limited, but current evidence suggests an autosomal dominant pattern of inheritance of nonsense mutations affecting *KRT14*, which codes for type I keratin protein [120,122]. Histopathological examination demonstrates localized pigmentary incontinence within the dermis with clumps of melanin-filled perivascular macrophages, liquefactive degeneration of the basal layer, and marked absence of adnexal structures [123,124].

*Elias* et al. reported the first known case of DPR affecting the beard in a 38-year-old Syrian man [123]. The patient presented to the clinic with numerous nonscarring alopecic areas of the beard with widespread areas of brown reticulate hyperpigmentation. The patient reported areas of hyperpigmentation beginning at birth and decreasing after he turned 30 years old. Hyperpigmentation was noted in the neck, trunk, extremities, axilla, and palms. Dermoscopic findings associated with dermatopathia pigmentosa reticularis include decreased hair density with fine vellus hairs, brown pigment globules, perifollicular reticular areas, white dots, and hypo/hyperpigmented macules [125]. On histology, there were scattered melanophages in the papillary dermis, perivascular melanophages, melanophages with spindle and epithelioid morphology, and absent adnexal structures. There are no reported treatments for the alopecia associated with DPR, but this case report highlighted a rare cause of beard alopecia.

## 7. Chemotherapy Induced Beard Alopecia

A widely known side effect of chemotherapy is alopecia. This may occur days following the initiation of chemotherapy or, more likely, 2–3 weeks after [126]. The pathophysiology of chemotherapy-induced alopecia is rooted in its attack on rapidly dividing cells, which involves the arrest of the anagen phase of the hair cycle resulting in the loss of dystrophic hairs, also known as anagen effluvium. The extent of hair loss depends not only on the potency and duration of the drug, but also on the phase of the hair cycle in which the hair follicle is in, the presence of underlying hair disorders such as AGA, and the type of hair. Scalp and beard hairs likely have the fastest rate of hair growth and are thus prone to the toxic effects of chemotherapy [127]. An anecdotal report from *Rebora* and *Guarrera* details decreased black and white beard hair density following treatment with docetaxel for approximately 2 months, but most white hairs continued to grow throughout therapy. However, once plucked, they were found to be dystrophic hairs. This may be due to white hairs’ broader shafts when compared to pigmented hairs which prevent the whole shaft from being affected, thus decreasing hair dystrophy [127]. In a study by *Chiewchanvit* et al. investigating mucocutaneous complications of chemotherapy in 74 Thai patients, 77.8% of patients experienced beard hair loss, with one report of beard hair pigment changes with therapy [128]. Early dermoscopy findings of chemotherapy-induced alopecia generally include black dots, broken hairs, exclamation mark hairs, flame hairs, follicle miniaturization, and pohl pinkus. Later findings include yellow dots, vellus-like hairs, pigtail hairs, and eventually, scattered regrowing hairs which appear as circle hairs with possible pigment abnormalities [126]. Chemotherapy-induced alopecia typically reverses in patients following cessation of therapy; however, some patients experience permanent or persistent alopecia or experience a change in hair features such as pigmentation and texture [126,127]. The only FDA-approved preventative treatment for chemotherapy-induced alopecia is scalp cooling. Minoxidil and prostaglandin analogs may be used after cessation of therapy to promote hair regrowth [129].

## 8. Non-Chemotherapeutic Drugs Causing Beard Alopecia

### 8.1. Ocrelizumab

Ocrelizumab is an anti-CD20 humanized monoclonal antibody used in the treatment of multiple sclerosis. There are multiple reports of alopecia areata as a side effect of various monoclonal antibody therapies; however, a case report by *Chin and AbduHilal* reports a rare finding of beard-only alopecia associated with ocrelizumab use in a patient with relapsing–remitting multiple sclerosis. While it is possible that AA onset may be distinct from medication use, according to the authors, its onset within 3 months of medication initiation makes this less likely [130]. Trichoscopic and histologic findings were not noted. The pathogenesis of this side effect is not fully understood, but it is hypothesized that AA induced by monoclonal antibody treatment may be due to the reprogramming of immune cells leading to decreased self-tolerance and therefore secondary autoimmunity [131]. Successful resolution was achieved with combined intralesional triamcinolone acetate, topical betamethasone valerate, and 5% minoxidil foam [130].

### 8.2. Anti-Parkinsonian Medications

Anti-parkinsonian medications have been reported to induce alopecia. A report by *Grauer* and *Sieb* describes a rare side effect of beard-only alopecia areata associated with combination cabergoline/selegiline within 6 months of medication initiation. Examination revealed exclamation point hairs, lack of pruritis, and lack of pigmentation and scaling [132]. Specific trichoscopic and histopathologic findings were not noted. The exact pathophysiology of this association remains unclear. An in vitro study by *Langan* et al. sought to characterize the effects of dopamine on hair follicles. The authors found that dopamine increased the percentage of hair follicles in the catagen phase and caused premature anagen termination in female hair follicles, suggesting that dopamine stunts hair growth and may cause hair loss. However, the exact mechanism is unclear, and the study’s clinical application is limited given that it is unknown how much dopamine actually reaches hair follicles in vivo with dopamine therapy [133]. While there is no targeted treatment for this side effect, dose reduction and medication discontinuation proved to reverse hair loss in some cases [134,135,136].

### 8.3. HIV Medications

Other drug-induced causes of alopecia have been reported in HIV patients undergoing treatment with antiretroviral medications [137,138,139,140]. Although it can be challenging to identify antiretroviral therapy as the causative agent of alopecia in this patient population due to possible confounders, it is important for clinicians to keep a high index of suspicion as this side effect has been reported in the literature, including beard hair alopecia [137,138,139,140]. Hair loss may be diffuse or patchy alopecia areata-like or may involve thinning and lightening of hairs causing the appearance of hair loss [137]. Protease inhibitors, such as indinavir, are most likely to cause hair loss in patients, commonly telogen effluvium [140]. Other side effects of indinavir therapy that may accompany alopecia include retinoid-like side effects such as cheilitis, dry skin, nail changes, and pyogenic granulomas [137]. This may be due to the upregulation of retinoic acid-related mechanisms by indinavir/ritonavir therapy, or rapid immune repopulation with antiretroviral therapy resulting in autoimmunity [137,139]. Histopathology may show hair follicles in the mid and upper dermis, consistent with presence in the telogen phase [137]. Discontinuation of the offending agent and/or alteration of the medication regimen may reverse hair loss in some cases [137,140].

## 9. Beard Alopecia Following Cosmetic Procedures

Deoxycholic acid (DA) injection is used to decrease preplatysmal fat and facial fullness by disrupting adipocyte cellular membranes [141]. DA injection ultimately leads to adipocyte cell lysis and decreases the number of adipocytes, reducing the appearance of facial fullness [141]. Common adverse effects of DA injection include injection site pain, swelling, and bruising [142]. Alopecia following DA injection is not listed as a major complication; however, increasing evidence has shown the potential for necrosis and temporary alopecia in the area of administration [143]. Three studies highlight temporary alopecia in the submental area of men following DA injection [144,145,146]. Biopsy findings reported in one case included elevated telogen–catagen hair count, some pigmented hair casts, maintenance of sebaceous glands, focal perifollicular lymphohistiocytic infiltrate in the dermis, and one subcutaneous follicle in the setting of fat necrosis with no peribulbar inflammation [146]. In most cases, the alopecia resolved after an extended period of time ranging from 7 to 14 months after DA injection [143]. *Souyoul* et al. treated their patient with topical 0.03% bimatoprost solution daily; however, the patient reported small areas of residual alopecia in the submental area eleven months after the initial DA injection [145]. Other reports have found that spontaneous regrowth of hair occurred without intervention months after DA injection [143]. Botulinum toxin injection has also been associated rarely with alopecia of the beard [147].

Given the potential psychosocial implications of beard hair appearance, there has been interest in investigating medications and procedures which enhance beard hair growth overall. In some studies, topical minoxidil has been shown to be a well-tolerated and clinically effective agent in stimulating beard hair growth [148,149]. Topical tretinoin has also been anecdotally shown to increase beard hair density, possibly owing to a high number of retinoic acid-binding proteins in hair follicle cells and a tretinoin-induced increase in growth factors and epithelium differentiation [150]. In patients with beta thalassemia major and associated hypogonadism, the application of testosterone gel significantly increased beard terminal hair number when compared to a control [151]. Topical latanoprost, with or without the addition of topical betamethasone valerate, has also been shown to stimulate beard hair growth in patients with AA [152]. Additionally, camouflage agents such as topical hair filler fiber have been shown to elicit high patient satisfaction in patients with alopecia; therefore, this technique could potentially be used to minimize the appearance of beard hair loss as well [153]. PRP, fractional carbon dioxide laser therapy, and microneedling are all treatments postulated to enhance hair growth, and a study by *Ragab* et al. compared the effects of PRP intradermal injection, fractional laser followed by topical PRP, and microneedling followed by topical PRP therapies on hair regrowth in alopecia areata. The authors found favorable, comparable results between all treatment modalities, with lesions on the beard showing the most pronounced improvement with therapy [154]. Lastly, more invasive procedures such as micropigmentation and hair transplantation may also enhance beard hair appearance [155,156].

## 10. Conclusions

Hair growth involves various mechanisms requiring a delicate interplay between numerous biological cues and cutaneous organs. Therefore, it is susceptible to dysregulation by several biological and environmental influences, including trauma, autoimmune dysfunction, infection, drugs, and endocrine disorders. In men, facial hair can serve as a symbol of masculinity and even a method of self-expression. Therefore, the possible negative psychosocial implications of hair loss in men should serve as an incentive for timely, accurate diagnosis and treatment.

This comprehensive review provides the clinician with numerous pathologies leading to beard alopecia and their potential respective treatments. Overall, timely diagnosis and treatment require a thorough history, including history of systemic inflammatory disorders, hair grooming techniques, psychological symptoms, infectious contact, medications, and lifestyle habits. Gross inspection of the lesions can further elucidate initial differentials to consider which can then be confirmed by trichoscopy and/or biopsy. Treatment is etiology-specific, with some responding more robustly to treatment than others. Although various treatment modalities may have to be trialed, in the end, successful treatment can have a positive psychosocial impact on patients’ lives.

## Data Availability

Not applicable.
